# Specifics of Porous Polymer and Xenogeneic Matrices and of Bone Tissue Regeneration Related to Their Implantation into an Experimental Rabbit Defect

**DOI:** 10.3390/polym16081165

**Published:** 2024-04-20

**Authors:** Diana Ya. Aleynik, Oleg P. Zhivtscov, Vladimir V. Yudin, Roman S. Kovylin, Roman N. Komarov, Irina N. Charykova, Daria D. Linkova, Yulia P. Rubtsova, Maria S. Guseva, Tatyana I. Vasyagina, Alexander G. Morozov, Sergey A. Chesnokov, Marfa N. Egorikhina

**Affiliations:** 1 Federal State Budgetary Educational Institution of Higher Education, Privolzhsky Research Medical University of the Ministry of Health of the Russian Federation, Minin and Pozharsky Square 10/1, Nizhny Novgorod 603005, Russia; daleynik@yandex.ru (D.Y.A.); zhivtsovoleg@gmail.com (O.P.Z.); yudin@iomc.ras.ru (V.V.Y.); bagsnn@gmail.com (R.N.K.); irina-ch0709@yandex.ru (I.N.C.); linckovadaria@yandex.ru (D.D.L.); rubincherry@yandex.ru (Y.P.R.); guseva_m@pimunn.net (M.S.G.); vasyagina_t@pimunn.net (T.I.V.); sch@iomc.ras.ru (S.A.C.); 2 G. A. Razuvaev Institute of Organometallic Chemistry of Russian Academy of Sciences, Tropinina 49, Nizhny Novgorod 603950, Russiamorozov@iomc.ras.ru (A.G.M.)

**Keywords:** hybrid porous polymer, interconnected porosity, bone regeneration, bovine-derived xenograft, bone graft substitute, biocompatibility, experimental rabbit bone defect

## Abstract

This paper provides a study of two bone substitutes: a hybrid porous polymer and an osteoplastic matrix based on a bovine-derived xenograft. Both materials are porous, but their pore characteristics are different. The osteoplastic matrix has pores of 300–600 µm and the hybrid polymer has smaller pores, generally of 6–20 µm, but with some pores up to 100 µm across. SEM data confirmed the porometry results and demonstrated the different structures of the materials. Therefore, both materials were characterized by an interconnected porous structure and provided conditions for the adhesion and vital activity of human ASCs in vitro. In an experimental model of rabbit shin bone defect, it was shown that, during the 6-month observation period, neither of the materials caused negative reactions in the experimental animals. By the end of the observation period, restoration of the defects in animals in both groups was completed, and elements of both materials were preserved in the defect areas. Data from morphological examinations and CT data demonstrated that the rate of rabbit bone tissue regeneration with the hybrid polymer was comparable to that with the osteoplastic matrix. Therefore, the hybrid polymer has good potential for use in further research and improvement in biomedical applications.

## 1. Introduction

It is known that bone is the second most transplanted tissue after blood [1], and the need for bone grafts or their substitutes (osteoplastic materials) is constantly increasing. This is due to the globally increasing number of patients with diseases that are accompanied by bone tissue loss. According to the statistics, the number of such patients could reach 20 million per year [2]. The restoration of bone defects is performed using current surgical technologies that often require the application of osteoplastic materials. Depending on the location and size of the defect, grafts of various sizes, configurations, porosities, and strengths may be required.

Until now, autografts have been the gold standard for bone grafting. However, they cannot be widely used due to the limited stock of donor material, the need for additional surgical intervention, and the possible donor site pathologies associated with the tissue sampling, increasing the risks of inflammation, infection, bleeding, and pain [3].

The second most widespread types of material used for bone defect replacement are allografts [4]. Allografts are cheaper than autografts, but their osteoinductive properties are lower, as they are void of cellular components [5,6]. In the case of allografts, resorption, the chances of infection, and the deceleration of consolidation are higher [7,8]. Moreover, one should note that there are ethical and legal problems associated with tissue donation, which have led to a shortage of donor material.

Another common way to create materials for bone grafting is the formation of tissue substitutes based on xenotissues. Such materials can contain a lot of collagen and essential mineral components, as well as having sufficient strength and porosity. However, the use of xenotissues is not without the risk of immune reactions and potential risks of infection; they also require additional and sometimes complex processing, meaning that some researchers do not recommend the use of such materials [9,10,11,12].

The necessary requirements for ideal bone replacement materials are widely known: good osteoinductivity, osteoconductivity, angiogenic potential, and biocompatibility, as well as an easily controlled shape and size, suitability for specific defects as necessary, low cost, and a capacity for long-term storage [12]. Taking into account the above-mentioned disadvantages of auto-, allo-, and xenografts, in recent years, bone tissue engineering has been actively developing, in particular in relation to the creation and study of artificial materials. For a long time, metal implants, mainly made from various titanium compounds, have been widely and quite successfully used in modern traumatology, orthopedics, and dentistry [13,14]. Therefore, one area of bone tissue engineering is the research and development of various polymer materials for assisting in bone tissue regeneration [15,16,17].

The use of such polymers can guarantee the availability of osteoplastic materials with the required characteristics, and studies of porous materials based on biocompatible and biodegradable polylactic acid (PLA) [18,19], polycaprolactone (PCL) [20,21,22], and bioinert polyurethanes [23], polystyrenes [24], and polyacrylates [25,26] are ongoing. Potential has been seen for hybrid composite materials consisting of biodegradable and bioinert polymers that combine the best properties of each polymer type, as well as for composites based on combinations of polymers with other materials [27]. Polymers can be obtained in any quantity and of any preset size and shape and can also be used as ink for 3D printing. Such materials can be stored for a long time and generally do not require specialized storage conditions; their primary cost, too, can be insignificant.

It should be emphasized here that the most difficult task in developing polymers for biomedical applications is in ensuring their biocompatibility and ability to support cellular processes [28].

Sufficient porosity is a prerequisite property of any material if it is to be used successfully in the facilitation of cellular processes, such as cell adhesion, migration, and proliferation during bone remodeling. Sufficient porosity is a prerequisite for ensuring the oxygenation, the diffusion of nutrients to, and the removal of waste from cells [29,30]. It is known that a system of interconnected porous architecture is the typical characteristic of a native bone structure that ensures ideal in vivo conditions for cell functioning, as well as for the transmission of the numerous diverse signals influencing cell development and differentiation. Modern materials for bone grafting are being created on the basis of “biomimicry”, which requires consideration of the internal porosity of such materials, involving the formation of a structure of interconnected pores and reflecting the above-noted important characteristics of bone [31]. Moreover, surface roughness and the modulus of compression of each material are also of utmost importance [32]. The combination of interconnected pores of various sizes (micropores < 2 µm, mesopores 2–50 µm, macropores > 50 µm, and megapores > 100 µm) can ensure the circulation of gases and liquids for the supply of nutrients, as well as the possibility of the migration of various cell types, ranging from blood cells (lymphocytes, macrophages) to mesenchymal stem cells and osteoblasts. At the next stage of bone remodeling, a system of interconnected pores should allow the generation of new vessels and the subsequent replacement of the graft material with own-body tissue.

Our research group has developed a porous hybrid material based on a polyoligomer [33], which is intended for the restoration of bone defects. The resulting material has been characterized as having a wide distribution of pore sizes ranging from 1 to 100 µm (the average pore size is approximately 10 µm) and an open porosity of approximately 70%. The material is non-cytotoxic and allows the loading of polylactide at up to 0.44 g per 1 g of the polymer matrix.

A polyoligomer matrix was synthesized in line with a previously described method [33,34], based on a combination of methacrylic oligomers that allowed the formation of a porous cross-linked polymer matrix, resistant not only to organic solvents, but also to physiological environments [35]. The application of a polylactide layer to the surface of the porous polymer resulted in the formation of a stronger hybrid porous material. Therefore, this material combined a stable bioinert matrix and a covering layer of bioresorbable polymer. Although the polymer matrix had the porous structure required for the implant material, it was of low strength. This meant that the implants based on it were inappropriate for use in replacing bone tissue defects. Polylactide, a biocompatible polyester, is one of the most commonly used materials in a number of different implant types [36,37]. In general, these range from interference screws, shafts, and pins to plates for osteosynthesis [38]. Such products also include implants with porous structures [39], produced by the extrusion of polylactide thread. They are of different morphologies, but the size of the frame elements is in the hundreds of microns [40,41], determined by the implant material manufacturing technique. As mentioned above, the effective penetration of extracellular fluid into the implant, ensuring the ability for blood clot formation in the implant, the migration of cells from the surrounding tissues and their subsequent proliferation, differentiation, and neovascularization, can take place subject to the availability of pores (frame elements, including items made of diacrylic oligomers) ranging from 5 to 100 microns [42].

The application of the polylactide layer to the surface of the polyoligomer matrix with pores of 1–100 μm allowed for a significant increase in strength without compromising the pore characteristics of the material. This made it suitable as a replacement material in experimental bone tissue defects for in vivo models. The bioinert scaffold made the material stable, providing the time required for bone remodeling in the damaged area. The polylactide coating also ensured biocompatibility of the material, facilitating its integration into tissues in the defect area. Moreover, the low-molecular-weight, water-soluble hydrolysis products formed during the gradual degradation of the polylactide can stimulate cell proliferation, thereby ensuring tissue regeneration [43].

The purpose of this study was to assess the safety and biocompatibility of the hybrid polymer in the restoration of an experimental defect in rabbit shin bone and compare it with a conventional medical product based on xenogeneic bone material.

In these experiments, a material based on xenogeneic bone was chosen as a reference because such materials are widely used in modern traumatology, orthopedics, and vertebrology and are more accessible and convenient compared with autografts and allografts. The implants made from these materials can be accumulated and stored in tissue banks in any quantity and any size; therefore, they are always ready for use. Xenogeneic materials are not limited in volume and do not require additional surgical interventions, unlike autografts, nor are there any ethical and legal problems with donors, unlike those associated with allografts.

To evaluate the behavior of hybrid polymer implants in the body of an experimental animal and compare this with a known osteoplastic matrix, samples of materials were implanted into the bone defects of rabbits. Rabbits are commonly used as experimental animals for assessing the processes of osteogenesis, angiogenesis, immune response, resorption, and infection after the implantation of biomaterials [44,45,46]. This is due to the fact that the biomechanical characteristics of rabbit bones are similar to those of human bones [47], and the size of these animals is sufficient for the implantation of mass grafts to study the tissue reaction in the area of contact between the graft and the donor bed, as well as the characteristics of any sample reconstruction. To study the biocompatibility and safety of the hybrid polymer material and compare it with an osteoplastic matrix, the authors chose a model using a defect in the upper third of the anterior internal surface of the rabbit left shin bone.

## 2. Materials and Methods

**Prototype samples.** The hybrid polymer material for the study was obtained from a porous polyoligomer matrix by applying a layer of polylactide—PLA (Mn—2.3 × 10^4^ Da) to the surface of the pores. The atactic microstructure PLA was obtained using the ROP (Ring-Opening Polymerization) of a lactide monomer catalyzed by the calcium metal complex, as previously described [43]. The monomer source, derived from L-lactic acid using our proposed approach [48], was purified through recrystallization from anhydrous diethyl ether followed by vacuum sublimation (95 °C, 5 × 10^−2^ mbar). The content of the D-form in the mixture of stereoisomers after purification was determined to be 12% through the polarimetry conducted using an Automatic Polarimeter (Atago SAC-I, Saitama, Japan).

**Samples of the reference material.** The osteoplastic matrix “bioOST (Cardioplant, Penza, Russia) was used as a control material—a comparison material. The osteoplastic matrix “bioOST” is a registered medical product manufactured using original technology. Although the manufacturer did not disclose the details of the technology in the summary description, it was stated that the material was obtained from the purified sterile bone tissue of bullocks subject to strict veterinary control. The material was affordable, and has been widely and successfully used in a number of clinics in traumatology, orthopedics, and neurosurgery (https://cardioplant.ru/trauma/bioost; accessed on 9 April 2024).

To clarify any individual peculiarities of the materials, an in vitro comparative specification of the pore characteristics, some mechanics, the structure of both materials, and determination of the possibility of their interaction with surface-dependent cells (human adipose-derived stromal stem cells—human ASCs) was conducted before the start of the experimental study on animals.

The levels of residual free radicals in the hybrid porous polymer were determined in order to exclude the possibility of potentially damaging effects on cells.

**Study of the sample characteristics**. Samples of the experimental material and reference material were measured and photographed before the study. Then, fluorochrome staining (Hoechst 33342, BD Biosciences™, Franklin Lakes, NJ, USA), excitation wavelength—377 nm and emission wavelength—447 nm) of the samples was performed both to confirm that there was no residual foreign DNA in the osteoplastic matrix and to visualize the samples’ pores. For this purpose, standard size samples of the materials were placed in the wells of a 24-well plate, each filled with 2 mL of growth medium and left for 1 h. Fluorochrome was used according to the manufacturer's protocol. 1 μL of Hoechst 33342 solution (concentration 1 μg/mL) was added to each well, and the plate with samples was put into a CO_2_ incubator at 37 °C, 5% CO_2_, and high humidity and left for 30 min. After 30 min of staining, the appearance of the samples was assessed using wide-field fluorescence microscopy. The fluorescence microscopy allowed us to visualize their porous structure.

The pore characteristics of the materials were determined using mercury porometry using a PASCAL EVO 140/440 ULTRA MACRO porosimeter (Thermo Fisher Scientific S.p.A., Rodano, MI, Italy). The scanning electron microscopy (SEM) studies of the material chips were conducted on a Regulus SU8100 microscope (Hitachi, Tokyo, Japan) at an accelerating voltage of 0,7 kV without applying a conductive coating.

The mechanical characteristics of the materials were determined using an Autograph AGX-V 50 universal testing machine with a 5 kN load cell (Shimadzu, Kyoto, Japan) at a deformation rate of 5 mm/min. The compression modulus was calculated using the slope of the linear range. The strength characteristics (Young's modulus in compression (E) and compression failure stress (σ)) were studied according to ISO 604:2002 [49]. For this, the samples were cut into 10 × 10 × 4 mm blocks using a blade.

The electron spin resonance spectroscopy (ESR) technique was used to monitor the residual free radicals in the hybrid porous polymer. For this purpose, a sample of finely dispersed hybrid porous polymer was placed in a glass tube with a diameter of 1.5 mm and the ESR spectrum was recorded using a Bruker Magnettech MS5000 (Bruker, Berlin, Germanya) spectrometer at room temperature: field center B0 = 338 mT (gi~2.0030); sweep value B = 12 mT; and modulation value = 0.2 mT.

**Study of the interaction of the human ASCs with the analysis samples in vitro.** To assess the adhesion and viability of the human ASCs on the samples of the materials used, human ASCs of passage 3 were seeded onto the surface of the experimental and control samples at a concentration of 20 × 10^3^ and cultured in a CO_2_ incubator under standard conditions (37 °C, 5% CO_2_, high humidity). 24, 48, and 72 h after sub-culturing onto the samples, the cells were stained with fluorochromes and video-recorded, and the cells on the surfaces of the samples were assessed.

Fluorescent staining of the cell nuclei was conducted using intravital staining with the fluorochrome Hoechst 33342 (USA, excitation wavelength 377 nm, emission wavelength 447 nm). To characterize viable cells that had adhered to the material, and their morphology, the cytoplasm was intravitally stained with Calcein AM (BD Pharmingen™ Franklin Lakes, NJ, USA). The principle of action of this dye is associated with esterase activity that is typical only of viable cells (excitation wavelength 495 nm, emission wavelength 515 nm). The fluorochrome staining was performed according to the manufacturers' protocols.

Fluorescence microscopy was implemented using the Cytation 5 imager (BioTek, Winooski, VE, USA) and Gen 5 Imedge+ software (https://www.agilent.com/en/product/cell-analysis/cell-imaging-microscopy/cell-imaging-microscopy-software/biotek-gen5-software-for-imaging-microscopy-1623226; accessed on 9 April 2024).

The test culture of the human ASCs for the study was obtained and characterized in the biotechnology laboratory of the Federal State Budgetary Educational Institution of Higher Education, the “Privolzhsky Research Medical University” of the Ministry of Health of the Russian Federation.

**In vivo experimental model**. The study protocol was approved by the Ethical Committee of the Privolzhsky Research Medical University of the Ministry of Health of the Russian Federation (N. Novgorod) No. 01 dated 21 January 2022. All procedures using animals were conducted in a vivarium in compliance with the requirements of the European Convention for the Protection of Vertebrate Animals Used for Experimental and Other Scientific Purposes (Strasbourg, 2006).

**Experiment**. The experiment was conducted on 30 mature male rabbits of the Grey Giant breed aged 6–8 months. The weights of the animals ranged from 3.2 kg to 3.8 kg. Two weeks before the start of the study, the animals were acclimatized and kept throughout the study in the vivarium of the Federal State Budgetary Educational Institution of Higher Education, the “Privolzhsky Research Medical University” of the Ministry of Health of the Russian Federation, which complied with the SPF (Specified Pathogen Free) standard. The animals were on a standard diet and drank water ad libitum.

Pain was relieved using intramuscular injection of a mixture of 1.0 mL of XylaVeta (Pharmamagist Ltd., Budapest, Hungary) and 1.0 mL of Zoletil (Virbac Sante Animale, Carros, France); after haircutting and shaving, the surgical site was treated with Avansept antiseptic (LLC “Avansept Medical”, Moscow, Russia).

Identical surgical operations were performed, during which a standardized defect was created in the upper third of the anterior internal surface of the left shin bone. A cortical defect measuring 4 mm × 6 mm was made along the longitudinal axis in the center of the bone surface. The bottom of the defect consisted of two areas: proximally—cancellous bone tissue; and distally—the beginning of the intramedullary cavity.

The rabbits were divided into two groups. In the animals in the experimental group, the inflicted defect was replaced with the based porous material. In the animals in the control group, the defect was replaced with the xenogeneic osteoplastic matrix. During the introduction of the material samples into the bone defect, the samples were tightly adjusted to ensure a maximum contact area in the region of the defect, and then fixed with a bone suture using VICRYL thread (2-0, Ethicon, LLC, Puerto-Rico, USA). Before completion of the operation, careful hemostasis was ensured, as well as layer-by-layer suturing of the wound.

During the observation period after surgery, the authors assessed the behavior of the experimental animals, changes in their appetite, weight gain, and the state of the tissues in the area of the surgical wound. All animals had free access to water and received sufficient food.

After 1 month, five animals from each of the experimental and control groups were removed from the experiment; 2 months after the operation, another five animals from each of the experimental and control groups were removed; the remaining animals were removed 6 months after the operation.

Before removal on the control days, the animals underwent a computed tomography (CT) examination (using the Toshiba 32 Aquillon scanner, Toshiba, Minato, Japan) with CT densitometry of the center of the graft and calculation of the Hounsfield index (an indicator of the density of the X-ray shadow).

The animals were removed from the experiment by air embolism under anesthesia within the specified timeframe. After removal, a fragment of the shin bone tissue of 15 mm thickness and up to 30 mm in diameter, including intact areas and the area of injury, was explanted from each test animal of both groups for morphological examination.

For morphological examination, the material was fixed in a 10% neutral buffer solution of formalin (Labiko, Saint Petersburg, Russia) for 2 days. The decalcification of the bone tissue was conducted in a Softidek EDTA-based decalcifying solution in an acid buffer. After the decalcifying solution, the material was washed with running water for 30–60 min. Then, it was histologically processed according to the standard scheme. After processing in ethanol, the material was placed into Gistomix Extra paraffin medium (OOO BioVitrum, Saint Petersburg, Russia).

Sections of 7–10 µm were obtained from the paraffin blocks using a Leica RM 2245 microtome (Leica Biosystems, Nussloch, Germany), mounted on glass slides with an adhesive coating (poly-L-lysine), and dried overnight in a TS-1/80 SPU thermostat (SKTB SPU, Smolensk, Russia) at a temperature of 37 °C. Mayer's hematoxylin (BioVitrum, Russia) and eosin (LenReaktiv, Saint Petersburg, Russia) were used for the survey staining. The connective tissue elements were localized on the sections using a Van Gieson staining kit (BioVitrum, Russia). A glasseal mounting medium (Labiko, Russia) was used to mount the sections under the coverslips.

Morphological assessment and photo-recording of the experimental material were performed using a Leica DMLS light microscope (Leica Microsystems, Wetzlar, Germany) with a working magnification range of 40 to 1000 times.

## 3. Results

**Figure 1:**
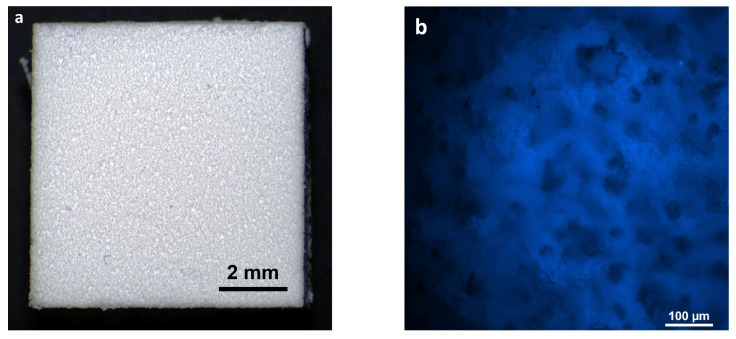
Hybrid polymer before starting the study: (**a**) external view of the sample; (**b**) porous structure of the sample before the experiment, fluorochrome Hoechst 3334, BD Pharmingen™.

**Characteristics of the hybrid polymer and osteoplastic matrix samples.** The samples were solid, opaque, white blocks (Figure 1a). Therefore, staining with Hoechst fluorochrome and observation at a high magnification ensured clear visualization of each sample’s porous structure (Figure 1b). The sizes of the samples corresponded to the sizes of the expected defects in the test animals.

**Figure 2:**
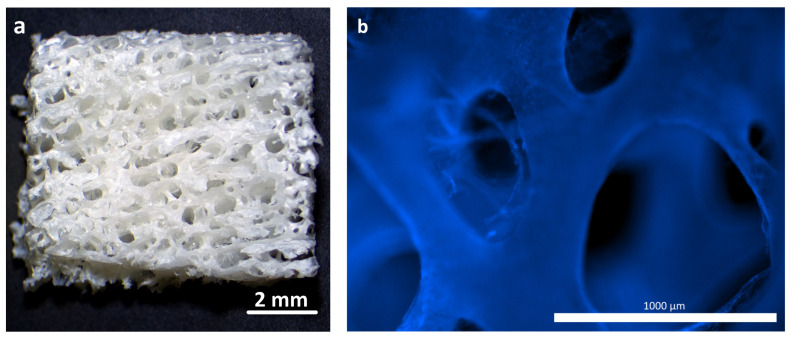
Osteoplastic matrix before starting the study: (**a**) external view of the sample; (**b**) porous structure of the sample before the experiment, fluorochrome Hoechst 3334, BD Pharmingen™.

**Control samples (material for comparison)**. The samples of the reference material (osteoplastic matrix) were white, hard blocks, with pores visible to the eye (Figure 2a). The Hoechst fluorochrome microscopy of the material (magnification 50×) showed the absence of residual DNA (no cell nuclei were detected in the material structure), while the porous structure of the material was clearly visualized (Figure 2b).

During the determination of the mechanical characteristics of the materials, the hybrid polymer material was demonstrated to have an elastic modulus of E = 80 MPa, and a compression failure stress of σ = 5.8 MPa, while the corresponding values for the reference material were: elastic modulus of E = 47.7 MPa, and compression failure stress of σ = 5.2 MPa.

The most important feature of the studied samples was their interconnected porosity. Some of the pore characteristics of the materials were determined using the mercury porometry method (Table 1).

**Figure 3:**
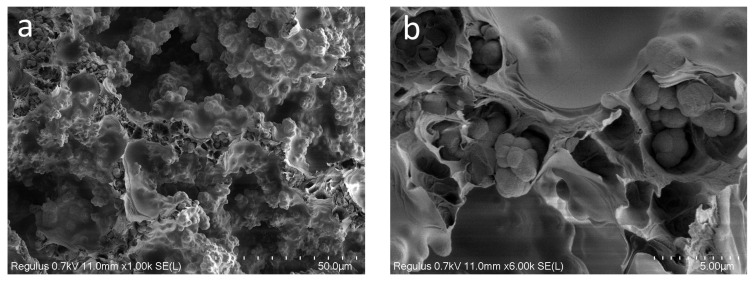
SEM images of the hybrid porous polymer: (**a**) 1000×; (**b**) 6000×.

The structure of the materials was studied using scanning electron microscopy (SEM). Images of sections of the hybrid porous polymer materials obtained using SEM are shown in Figure 3a,b. It can be seen that the structure of the sample formed a unified system of interconnected agglomerates of spherical particles that were 0.5–1 μm in size, looking similar to a coral. The size of the agglomerates reached 3–5 microns. The size of the agglomerates could be flexibly determined and adjusted by the conditions of the synthesis of the porous polymer, that is, by the composition of the polymerizing compound, the temperature, and the intensity of the initiating radiation. These polymer agglomerates were coated with a polylactide (PLA) film that made the surface of the globules smoother. It was also clear from the obtained microphotographs that the thickness of the PLA film was 0.1–0.2 μm, and that the macropores were not closed by the polylactide; the system of interconnected open pores was preserved. Overall, the unified system of the interconnected agglomerates of spherical particles formed pore walls of various shapes with a diameter of ~10–12 μm, which was consistent with the results of the mercury porometry.

**Figure 4:**
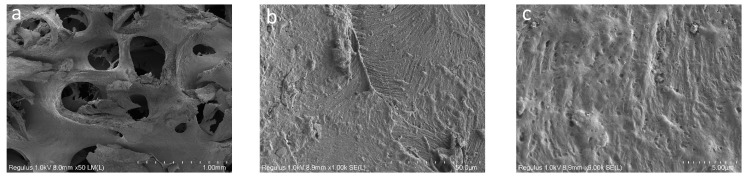
SEM images of osteoplastic matrix: (**a**) 50×; (**b**) 1000×; (**c**) 6000×.

The SEM images of the osteoplastic matrix looked different (Figure 4). It can be seen that the osteoplastic matrix was a structure with interconnected large cavities with a diameter ranging from 100 to 600 µm; the walls of the cavities were more compact compared with those of the hybrid polymer.

As specified, both materials were characterized by a system of interconnected pores, but they were different in their pore characteristics. For instance, the total porosity of the hybrid polymer was 66.9% (open porosity—47.8%), and the total porosity of the osteoplastic matrix was 78% (open porosity—75%); the average pore sizes were 17.5 μm and 567.9 μm, respectively.

Thus, overall, the structures of the materials were somewhat different. The osteoplastic matrix was represented by a large pore structure, the pore walls of which were rough, but more monolithic compared with the experimental material. The pore surface structure of the hybrid polymer was globular and formed by interconnected small agglomerates of spherical particles.

**Figure 5:**
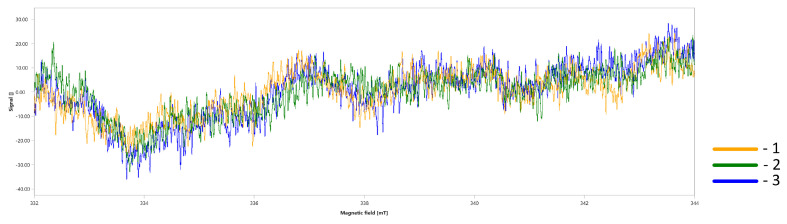
ESR spectra of the empty glass tube: (1) glass tube with a hybrid material sample in air; (2) and under vacuum; (3) Tube diameter 1.5 mm; T = 298 K. Recording parameters: field center B0 = 338 mT (gi~2.0030), sweep value B = 12 mT, modulation value = 0.2 mT.

When monitoring the residual free radicals, the ESR spectra of an empty tube, a tube with a sample, and a tube with a vacuumed sample were obtained; these spectra were identical, indicating the absence of free radicals in the studied sample (Figure 5).

**Interaction of human ASCs with the hybrid porous polymer and the control material samples.**

**Figure 6:**
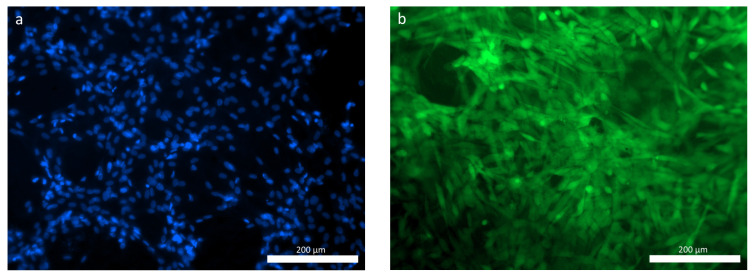
ASCs on the hybrid polymer, 72 h cultivation: (**a**) cell nuclei stained with fluorochrome: Hoechst 3334, BD Pharmingen™; (**b**) viable ASCs, cytoplasm stained with Calcein AM fluorochrome (BD Pharmingen™).

When studying the ASCs during cultivation using fluorescence microscopy, significant numbers of the human MSC-AT cells were attached to the surface of the hybrid polymer samples. These were spread out over the course of the 3 days (72 h) after seeding onto the samples, maintaining their typical morphology and viability (Figure 6a,b). The figure demonstrates that, given the small pore size of the material, the largest proportion of cells was located on the surface of the sample, in accordance with its topography (Figure 6b).

**Figure 7:**
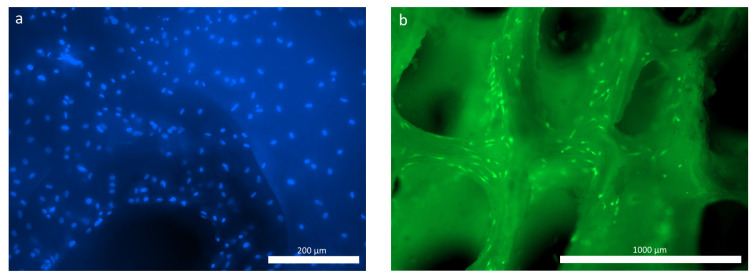
ASCs on the osteoplastic matrix, 72 h cultivation: (**a**) cell nuclei stained with fluorochrome: Hoechst 3334, BD Pharmingen™; (**b**) viable ASCs, cytoplasm stained with Calcein AM fluorochrome (BD Pharmingen™).

As for the osteoplastic matrix samples, after 24 h of cultivation, many cells were already fixed within the pores of the samples, although some could be visualized spread out on the surface. By 72 h of observation, viable calcein-stained cells of a typical fibroblast-like shape with pronounced processes were visualized on the surfaces of the osteoplastic matrix samples (Figure 7b).

The lack of cytotoxicity, good adhesion, spreading with the formation of a typical morphology, and the maintenance of viability of the test culture cells on the surface of the samples enabled us to conclude that both materials can provide favorable conditions for cellular processes when used as bone tissue substitutes; therefore, they can be applied in further research on experimental in vivo models.

**State of experimental animals during observation.** All experimental animals underwent surgery satisfactorily, remained active during the postoperative observation period, and gained weight. No infectious diseases or complications of a general or local nature were detected.

**Figure 8:**
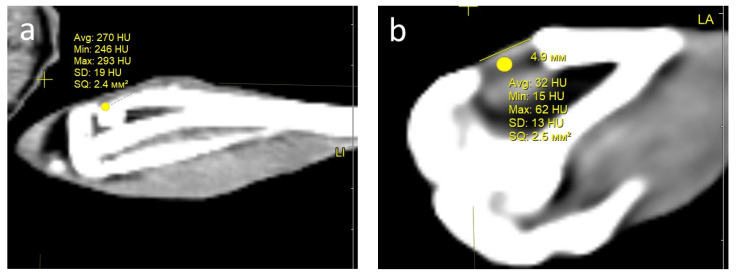

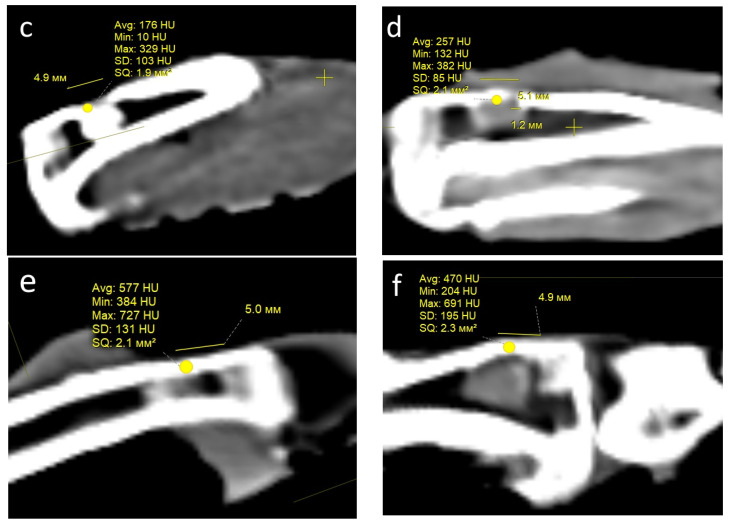
CT scan—section through the center of the implant in the sagittal direction, observed: at 1 month: (**a**) control, (**b**) experiment; at 2 months: (**c**) control, (**d**) experiment; at 6 months: (**e**) control; (**f**) experiment.

**Condition of the defect area and computed tomography (CT) examination**. One month after the operation, all rabbits in both groups showed signs of regeneration of the inflicted experimental bone defect in the form of a decrease in its size by 1–2 mm. The size of the defect, determined by CT measurements on the day of surgery, corresponded to the intraoperative measurements. At the CT densitometry assessment of the area after 1 month, the Hounsfield index among all rabbits in the control group (grafted osteoplastic matrix) was 69–271 HU. The average values of the X-ray density of the entire volume of the graft in the animals in this group were 142–229 HU, which indicated the heterogeneous nature of the cavity contents (Figure 8a). The values of this parameter in the experimental group (hybrid polymer) ranged from 15–89 HU. The average values of the X-ray density of the entire volume of the graft in the animals in this group were 31–67 HU (Figure 8b). This very insignificant scattering of values indicated the homogeneity of the graft. In the cortical bone structures adjacent to the defect, the Hounsfield index value was 680–720 HU.

In the animals removed after 2 months, a further decrease in the size of the defect was seen, decreasing to an insignificant size of 1–2 mm. All animals in the control group (grafted osteoplastic matrix) had absolute values of the Hounsfield index from 10–457 HU. The average values of the X-ray density of the entire volume of the graft in the animals in this group were 171–231 HU (Figure 8c). The minimum value in the experimental group (hybrid polymer) was 96 HU, the maximum was 643 H. The average values ranged from 195–453 HU (Figure 8d). The measurements did not reveal any pathological changes in the cortical bone structures adjacent to the defects.

After 6 months, complete recovery of the applied defect was demonstrated in all animals in both groups. However, the Hounsfield index in the control group (grafted osteoplastic matrix) ranged from −60 to 910 HU and continued to demonstrate the heterogeneity of the graft tissue, along with occasional increases above the usual values for shin bone. The average values ranged from 305–577 HU (Figure 8e). The values of the X-ray density of the central part of the graft in the experimental group (hybrid polymer) ranged from 204–814 HU. The average values ranged from 426–615 HU (Figure 8f).

The dynamics of the changes in X-ray density in the defect area are shown in Table 2.

Therefore, the CT data, which includes CT densitometry of the central area of the graft, indicate that the processes of reparative regeneration in the animals in the control and experimental groups differed to some extent. In the control group, at all assessment points, the density of the graft was convincingly heterogeneous, and included areas where minimal X-ray shadow intensity persisted for 6 months. The graft placed into the bone defect in the rabbits in the experimental group demonstrated a stable increase in X-ray density, together with a less significant scatter between the minimum and maximum values of the X-ray shadow intensity.

Consequently, based on the CT image analysis, the authors determined the development of a more uniform structure; therefore, it was concluded that there were more active regenerative processes in the bone defects of the rabbits in the experimental group.

**Morphological examination**. During the histological analysis after 1 month of observation, the area of the modeled defect could clearly be determined in the rabbits in both groups, which corresponded to the visual picture and CT data.

**Figure 9:**
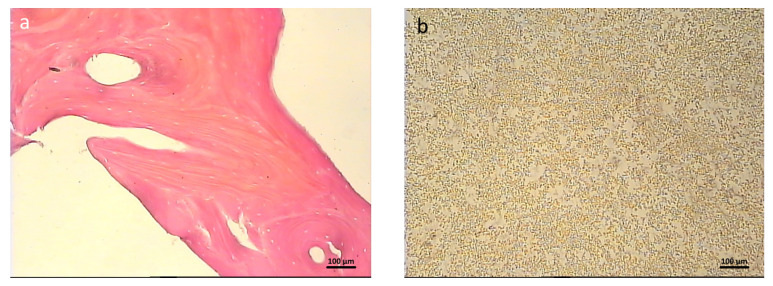
Microstructure of materials, Van Gieson staining, 100×: (**a**) osteoplastic matrix; (**b**) hybrid polymer.

**Figure 10:**
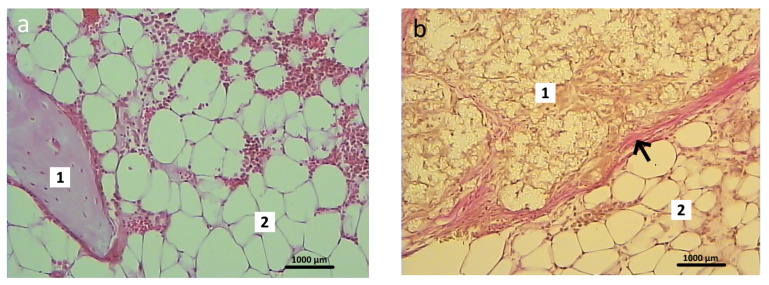
Graft localization area in animals 1 month after surgery: (**a**) control group (1—fragment of the control material–osteoplastic matrix, 2—bone marrow). Hematoxylin and eosin staining; 200×); (**b**) experimental group (1—elements of experimental material–hybrid polymer; 2—bone marrow; connective tissue fibers are indicated by arrows). Van Gieson staining; 200×.

In the animals in the control group, which had had the osteoplastic matrix implanted in the bone defect area, the graft localization area had clear contours on this section. The control graft structurally resembled cancellous bone tissue with well-developed trabeculae, which were stained pink or red depending on the dye applied (Figure 9a). The control graft was tightly attached to the bone marrow elements (Figure 10a). The perifocal area of the animals in the control group was characterized by signs of periosteal hypertrophy and areas with foci of chondrocyte-like, poorly differentiated cells. The bone defect area was separated from the surrounding tissues by a dense layer of connective tissue.

In the bone tissue samples of the animals in the experimental group, after 1 month of the postoperative period, the entire volume of the bone defect was filled with the hybrid polymer material. This had an amorphous structure and was almost unstained by the histological dyes (Figure 9b). The sections showed that the material adhered tightly to the bone fragments. The experimental sample was separated from the bone marrow elements by a narrow strip of loose fibrous connective tissue (Figure 10b), which also extended to cover the bone defect area.

**Figure 11:**
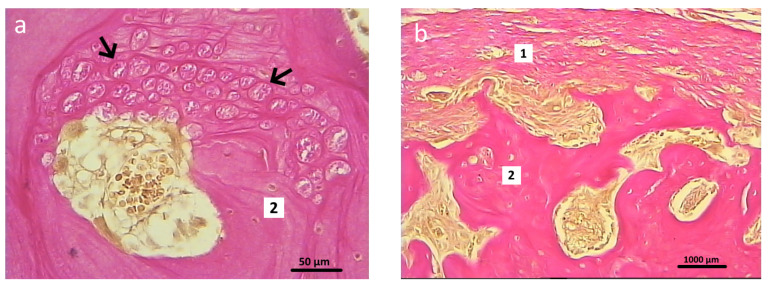
Perifocal area in the experimental group animals 1 month after surgery: (**a**) accumulation of chondrocyte-like cells (indicated by arrows) in close contact with elements of the bone tissue—2, Van Gieson staining: 400×; (**b**) erosion lacunae in the form of clefts are clearly visible in the contact area of the periosteum inner layer—1 and bone compact substance—2. Van Gieson staining; 200×.

The perifocal area, as with that in the control group, contained areas filled with chondroid-type cells (Figure 11a). Erosion lacunae in the form of clefts were clearly visible in the area of contact between the compact bone substance and the inner layer of the periosteum. These lacunae occurred both in isolation and accumulated in large groups (Figure 11b). There were no signs of any inflammatory reaction in either the control animals or the experimental group animals.

**Figure 12:**
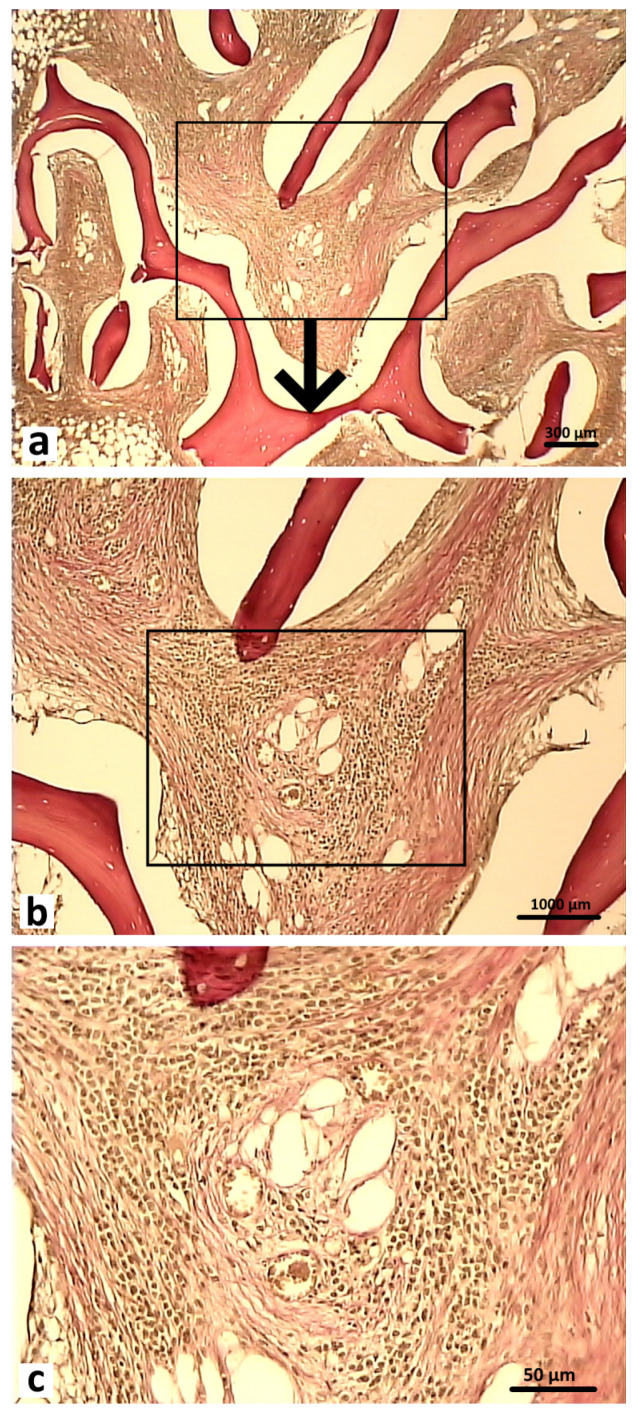
Graft localization area in the control group animals 2 months after surgery. Structural elements of the osteoplastic matrix surrounded by the heterogeneous cell-fiber population of the fibroreticular tissue. Functionally active microvessels (indicated by arrows), Van Gieson staining: (**a**) 40×; (**b**) 200×; (**c**) 400×.

After 2 months of the postoperative period, differences were noted in the signs of reparative regeneration in the graft localization area between the control and experimental group animals. The process of integration of the control material into the surrounding tissues was more intensive. These sections showed structural elements of the osteoplastic matrix, surrounded by a heterogeneous cell-fiber population of fibroreticular tissue and functionally active microvessels (Figure 12).

**Figure 13:**
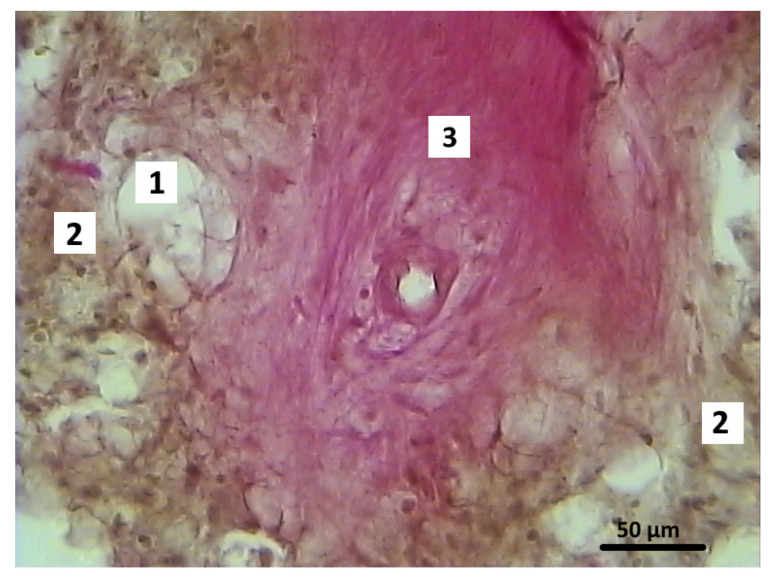
Graft localization area (hybrid polymer) in animals in the experimental group, 2 months after surgery: 1—experimental material; 2—poorly differentiated cells; 3—connective tissue fibers. Van Gieson staining; 400×.

After 2 months of the postoperative period, the sections of bone tissue in the experimental group animals demonstrated poorly differentiated groups of cells and connective tissue fibers, and single microvessels in the graft localization area (Figure 13).

**Figure 14:**
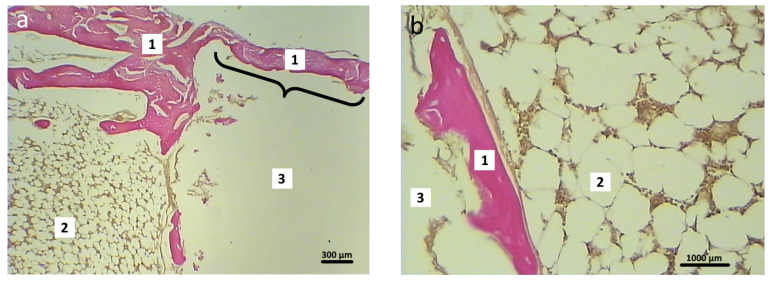
Graft localization area (hybrid polymer) in animals in the experimental group, 2 months after surgery (1—newly formed bone tissue, 2—bone marrow, 3—implant zone. The area of the bone defect is indicated by a curly bracket). Hematoxylin and eosin staining: (**a**) 40×; (**b**) 200×.

Newly formed bone tissue was found in the area of graft contact with the bone marrow and in the bone defect area in the animals in the experimental group (Figure 14). At 2 months after surgery, the foci of chondroid differentiation in the perifocal area remained in both the control and experimental group animals.

**Figure 15:**
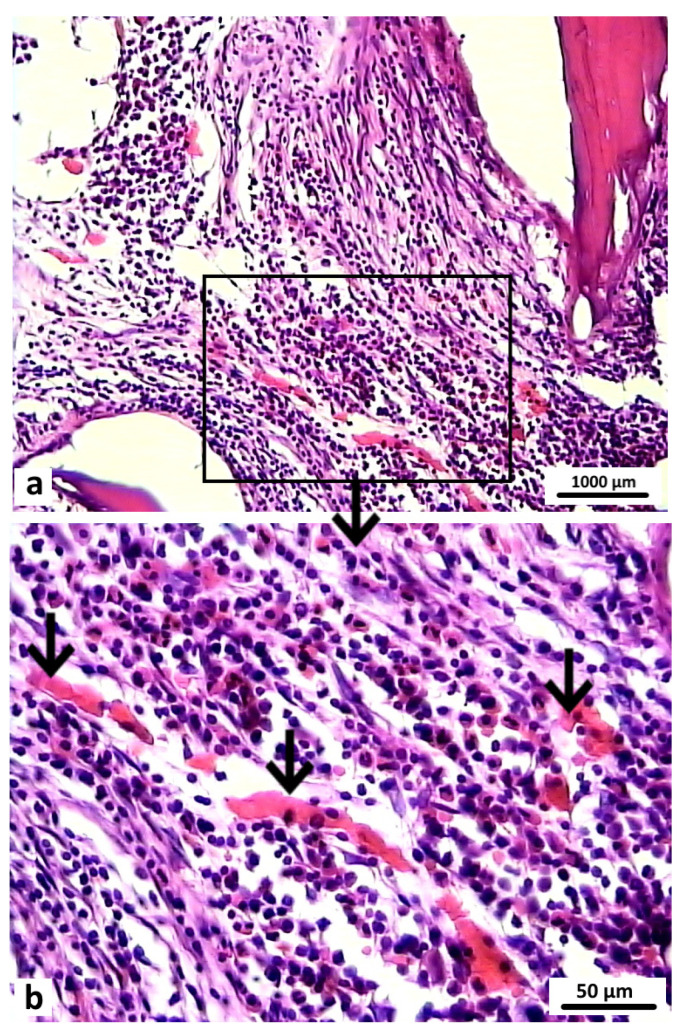
Area of osteoplastic matrix localization in animals in the control group 6 months after surgery. Reticulofibrous tissue with thin bone trabeculae (indicated by arrows). Hematoxylin and eosin staining: (**a**) 200×; (**b**) 400×.

After 6 months, the bone tissue samples of rabbits in the control group showed that the defect was completely filled with the bone regenerate and the surface was covered with a thickened periosteum. In the area of the bone defect bed, the regenerate was represented by fresh bone tissue. Functionally active osteoblasts were identified on the surface of the developing bone trabeculae. The structural elements of the osteoplastic matrix were preserved, but these were thinned and had signs of resorption. The graft localization area in these animals in the control group at 6 months after surgery was represented by reticulofibrous tissue with thin bone trabeculae (Figure 15).

**Figure 16:**
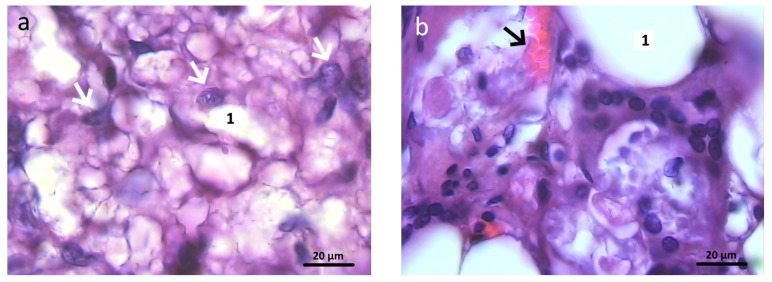
Graft localization area in animals in the experimental group 6 months after surgery (1—hybrid polymer), 1000×: (**a**) heterogeneous group of cells (indicated by white arrows); (**b**) microvessels (indicated by black arrows) in close contact with small particles of the hybrid polymer. Hematoxylin and eosin staining.

Histological preparations of bone tissue sampled from animals in the experimental group during this observation period showed that the defect was also completely covered by lamellar bone tissue. In the graft localization region, small areas filled with experimental material were found in close contact with heterogeneous groups of cells and microvessels (Figure 16).

Therefore, the data obtained in the morphological examination demonstrated that the rate of bone tissue regeneration observed with the hybrid polymer was comparable with the rate observed with the osteoplastic matrix. The results of this long-term investigation enable us to conclude that the graft samples made of hybrid polymer material were safe and biocompatible. Therefore, this material (PHB) can be used to replace bone defects to help optimize osteogenesis in the case of bone tissue damage. It can be assumed that the graft indirectly influences the process of aseptic inflammation in the wound and stimulates the proliferation of osteogenic cells.

## 4. Discussion

The authors conducted a comparative study of two different materials used in bone defect replacement: an osteoplastic matrix derived from xenogeneic bone and a hybrid polymer. Both materials were porous. As mentioned above, the material porosity, along with its mechanical properties, are critical to providing support and a platform for cell adhesion and nutrient exchange [50]. Pore size, in turn, affects cell proliferation and promotes the production of the extracellular matrix. There is no consensus regarding the optimal pore size; some experts consider the predominance of large pores (100–300 µm) optimal to ensure such cellular processes, while others have demonstrated the critical importance of micropores (10–60 µm) for such processes [40,51].

The materials used in this study differed in pore size: megapores (100–600 μm) were recorded in the osteoplastic matrix, whereas pores of 10–20 μm predominated in the hybrid polymer, along with significant numbers of 100 μm pores. As shown by Kim et al., when studying the effect of pore structure on bone regeneration in vivo, it is micropores (<25 μm) that can induce the maturation and remodeling of new bone [52].

However, when comparing the interaction of the ASCs with the samples, the authors noted that the cells adhered well, spread out, and remained viable on the surfaces of samples of both materials. Based on the data obtained during their interaction with the human ASCs in the in vivo model, it was concluded that both materials could contribute to supporting cellular processes.

The material used in this study consisted of a porous matrix coated with PLA. The PLA was used to cover the framework as it is a polymer with known biological characteristics. It is also biocompatible, biodegradable, and can provide appropriate conditions for cell attachment and subsequent tissue regeneration [53]. It is known that PLA has quite high mechanical characteristics [54] and can be used to replace bone tissue [55]. Therefore, the use of PLA in the development of the hybrid polymer allowed us to increase the mechanical strength of the framework, while ensuring partial, gradual biodegradation of the material, and, when necessary, the possibility of functionalizing the material with various substances, for example, to ensure long-term antibacterial activity [24]. PLA offers some advantages, including availability, low cost, and applicability for 3D printing, as demonstrated in a number of previous studies on trabecular bone replacement scaffolds [39,56,57,58].

When studying the peculiarities of the bone defect restoration using materials intended for bone grafting in experimental models, one should consider that these complex processes depend both on the material and on the experimental model: for example, the animal type and the location of the bone defect [58,59,60].

To assess the processes of rabbit bone tissue restoration, the authors evaluated the overall condition of the animals, in addition to using X-ray and CT examinations, and histological methods, on the defect areas.

The assessment of the changes in the histological pattern in the areas where the studied materials interacted with the bone tissue, above all, takes into account the signs of inflammation, the extent of vascularization, and the formation of new bone and its maturation, while assessing the state of the areas surrounding the grafts [61]. When studying such graft materials, one must understand how long their elements remain in the experimental defect area.

Usually, during studies of materials in experimental animals, researchers work on the principle that the duration of the experiment should be 3–4 months, because the graft must remain strong for at least 4 months. This is related to the fact that, in clinical practice, the range of periods for retaining a graft varies, depending on the site of implantation, the age, and on any pathologies of the recipient; on average, such impacts are evidenced within 4 months. However, it should be taken into account that bone tissue restoration includes several stages: the inflammatory stage (3–7 days), the recovery stage (3–4 months), and, finally, the stage of continuous remodeling, which can vary from months to several years [62]. During a sufficiently long stage of bone remodeling, the graft material should be able to remain in the reconstructed defect area without causing any disruption to the restoration process.

For example, by studying two different xenomaterials (based on bovine and porcine bones) in a rabbit bone defect model, Calvo-Guirado et al. (2015) showed that specific changes in the repair process were dependent on the nature of the material, even though both materials could be successfully used for bone grafting. By the end of the study (4 months), the elements of both implanted materials remained in the defect area, but these did not interfere with the restoration of the bone integrity [63].

Minto B.W. et al. (2021) used a rabbit radius bone model to study the restoration of a critical defect using a complex polymer matrix made of PLLA composite and hydroxyapatite powder, and compared the process of defect restoration with restoration using an autograft [64]. The researchers noted that, by the end of the observation period (90 days), graft material still remained in the defect area, but the process of bone integrity restoration was not disrupted. However, animals treated using the composite material had worse clinical and histological outcomes compared with the autograft group, which was expected and highlighted when using autologous materials [27,65].

P. Duan et al. [66] prepared two-layer PGLA scaffolds with various pore sizes in different layers and studied them in a rabbit model of an osteochondral defect on the articular surface of the femoral condyle. After 24 weeks (6 months), the scaffold elements could still be identified in the defect area. The PLGA material demonstrated good biocompatibility and processability. Moreover, it was proven that the bilayer PLGA scaffold could facilitate the simultaneous regeneration and repair of various tissues.

In this study, the authors deliberately chose a material based on xenogeneic bone and approved for clinical use as the control, for comparison. The experimental study in animals showed that, in this experimental model, both materials were safe, biocompatible, and were not rejected.

There were no signs of pathological inflammation during the histological examination at any of the study stages (1, 2, 6 months) in either group of animals, which is the most important evidence of the materials’ safety. Also, while elements of both materials remained in the defect area until the end of the study, as was found in previous studies, this still allowed the experimental defects to be completely restored in animals in both groups.

During the development and further adjustment of a new polymer material for use to help restore bone tissue, understanding the timeframe of the material’s retention in the body of the experimental animal is of utmost importance.

It is known that bone formation can be achieved in two ways: intramembranous ossification (IMO) and endochondral ossification (EO). These mechanisms are critical for the natural repair of bone after injury. IMO may increase the number of osteoblast-associated cells in the inner and outer periosteum, leading to the thickening and calcification of the periosteum and then to the connection of the fracture ends. EO mainly facilitates the sterile inflammatory reaction between the broken end hematoma and the bone marrow cavity with the surrounding environment, thus forming granulation tissue, fibrous tissue, and temporary cartilaginous tissue. In turn, osteoblasts penetrate and replace the chondrocytes, ultimately forming new bone tissue [67].

During the experiment, the authors noted that, in both cases—with the control xenogeneic material and with the hybrid porous polymer—starting from the early stages and during the period of up to 6 months of repair, chondrocytes could be recorded in the defect area. This may indicate a predominance of endochondral osteogenesis when using either material.

The results of the study did not reveal any significant differences in timing or in the visual characteristics of restoration of the experimental defects in the shin bones of rabbits during the implantation of the control and experimental bone replacement material. CT data and densitometry recorded a more stable and uniform increase in the X-ray density of the defect area in animals in the experimental group, whereas in the control group (grafted osteoplastic matrix), the density of the graft remained heterogeneous at all timepoints throughout the study. The data available indicated the development of a more uniform structural adjustment in the area of the surgical defect and, thus, more active regenerative processes in the bone defects of rabbits in the experimental group.

The morphological analysis data, in turn, confirmed the comparability of the rates of bone tissue regeneration with the graft made from the developed hybrid polymer, when using the control material. By the end of the study, the bone defects in animals in both groups were filled with fresh bone tissue, while elements of the graft materials were preserved in the areas of bone defects in animals of both groups by the end of the 6-month observation period.

Therefore, within the scope of this study, it was shown that during a chronic experiment (up to 6 months) in an animal model, the developed hybrid polymer was safe and biocompatible.

## 5. Conclusions

This comparative study of the hybrid polymer and the osteoplastic matrix showed that, despite the varied nature and differences in pore sizes, both materials were characterized by an interconnected porous structure that provided conditions for adhesion and the vital activity of human ASCs in vitro.

An in vivo experimental model showed that, by the end of the observation period (6 months), the restoration of the defects in animals in both groups was completed. Data from the morphological examination demonstrated that the rate of regeneration of rabbit bone tissue with the hybrid polymer was comparable with that using the osteoplastic matrix. Furthermore, CT data and densitometry measurements indicated the development of its impact on allowing a more uniform structural adjustment in the area of the surgical defect. Thus, the hybrid polymer demonstrated in vitro cytocompatibility, safety, and biocompatibility in the restoration of bone defects in the experimental animal model, which were similar to the characteristics of the registered osteoplastic matrix based on xenogeneic tissues. The data provided by this study allow us to consider the developed hybrid polymer as promising for further research and improvement, with a view to its use in biomedical applications.

## Figures and Tables

**Table 1:** Materials pore characteristics.

Parameters	Hybrid Polymer	Osteoplastic Matrix
Specific pore volume (V sp).	1.178 cm^2^/g	1.862 cm^2^/g
Pore size (D)	1–100 μm	80–750 μm
Apparent density (P app.)	0.405	0.403
Total porosity Ƹtotal.	66.9%	78%
Open porosity Ƹopen.	47.8%	75%

**Table 2:** Hounsfield index values (in HU).

Tests Deadlines	Groups of Animals		
		Control (Xenogeneic Material)	Experience (Hybrid Polymer)
1 month	Average values	142–229	31–67
	Min—Max	69–271	15–89
2 months	Average values	171–231	195–453
	Min—Max	10–457	96–643
6 months	Average values	305–577	426–615
	Min—Max	60–910	204–814

## Data Availability

The raw/processed data required to reproduce these findings cannot be shared at this time as the data also form part of an ongoing study.
